# SNP-based linkage mapping reveals novel quantitative trait loci for yield traits in noug (*Guizotia abyssinica* (L. f.) Cass.)

**DOI:** 10.3389/fpls.2025.1662582

**Published:** 2025-09-09

**Authors:** Adane Gebeyehu, Cecilia Hammenhag, Ramesh R. Vetukuri, Rodomiro Ortiz, Mulatu Geleta

**Affiliations:** Department of Plant Breeding, Swedish University of Agricultural Sciences, Alnarp, Sweden

**Keywords:** *Guizotia abyssinica*, candidate genes, comparative genomics, marker-assisted selection (MAS), QTL mapping, SNP markers

## Abstract

Noug (*Guizotia abyssinica*) is a vital Ethiopian oilseed crop lacking comprehensive genomic resources. This study constructed the first high-density SNP-based linkage map for this diploid species (2n=30, genome size ~1.7 Gb). Using an F_2_ mapping population of 286 individuals, we generated 13,888 high-quality SNPs from genotyping-by-sequencing (GBS), which were mapped onto 15 linkage groups (LGs) with a mean marker density of 2.1 cM, covering 90.6% of the genome. Phenotypic evaluation revealed significant variation for nine agronomic traits, including plant height (110–292 cm), days to flowering (49–115 days), and oil content (13.88–55.62%). Quantitative trait loci (QTL) mapping identified 27 QTL for six traits. Major findings include a flowering time QTL (qDTF-9-1) on LG9 explaining 7.6% of phenotypic variation (PVE) and a seed yield QTL (qNSPP-5-1) on LG5 explaining 2.9% PVE. Comparative genomics with sunflower (Helianthus annuus) revealed significant synteny, enabling the identification of candidate genes underlying these QTL: CLC-b (for qDTF-9-1) and GPT1 (for qNSPP-5-1). Additional QTL were detected for thousand-seed weight (cumulative PVE 51.2%), flower size (47.5%), capitula number (32.8%), and oil content (38.1%). This high-density genetic map and the identified QTL provide a foundational genomic resource for marker-assisted breeding to improve yield and agronomic traits in noug.

## 1 Introduction

Noug (*Guizotia abyssinica* L.) is Ethiopia’s second most important edible oilseed crop, cultivated mainly for its oil, while its seeds are also rich in protein (Geleta and Ortiz, 2013; Gebeyehu et al., 2021; Tsehay et al., 2021). It provides over 60% of the edible oil needs and supports the livelihoods of millions (Geleta et al., 2002; Geleta and Ortiz, 2013). It is cultivated in a total area of 358,828 hectares, with a total production of 295,000 MT (CSA, 2021; USDA-GAIN, 2021). Although primarily grown in Ethiopia and India, noug is also cultivated on a smaller scale in other African and Asian countries (Getinet and Sharma, 1996). Its oil content and fatty acid composition vary depending on seed maturity and geographic origin (Ayana et al., Gupta et al., 2017), with oil content ranging from 27% to 56% (Geleta et al., 2011). Linoleic acid comprises > 60% of the total fatty acids in noug oil (Dagne and Jonsson, 1997; Ramadan and Mörsel, 2003). Oleic, palmitic, stearic, and other unsaturated fatty acids constitute the remaining percentage. Depending on the genotype and environmental conditions, Ethiopian noug seed oil contains 51–80% linoleic acid (Petros et al., 2009; Geleta et al., 2011; Tsehay et al., 2021).

Currently, noug yields average only 0.8 to 1.2 tons per hectare, far below the 3.5 tons per hectare obtained in improved sunflower varieties (Diriba, 2018; CSA, 2021; USDA-GAIN, 2021). The rising population and climate change are projected to intensify temperature and rainfall variability across East Africa, reducing favorable cultivation areas for noug, underscoring the necessity of enhancing noug resilience and productivity (Gupta et al., 2017; Gebeyehu et al., 2021).

Despite its socio-economic relevance, noug is still one of the least genetically studied oilseed crops globally. While significant oilseeds, such as soybeans, rapeseed, and sunflowers, have had extensive genomic details, noug lacks even basic molecular tools to aid in genetic improvement. The gap is particularly telling given that noug cultivation in its center of origin predates several modern oilseed crops (Dempewolf et al., 2015; Ayana et al., 2016; Gupta et al., 2017). Additionally, the lack of marker-trait associations delays the adoption of molecular breeding techniques to enhance seed yield potential (Gupta et al., 2017; Zhang et al., 2018; Hammenhag et al., 2020). The current limitations in noug genomics present several challenges towards crop improvement. Conventional breeding methods have limited success in selecting for traits such as drought tolerance and disease resistance due to the lack of genomic information, even though studies have highlighted noug’s susceptibility to abiotic stresses and fungal pathogens (Riley and Belayneh, 1989; Dutta et al., 1994; Getinet and Sharma, 1996; Ayana et al., 2016; Gupta et al., 2017). While some initial steps have been taken, such as the development of transcriptome-based SNP markers by Tsehay et al. (2020), a comprehensive linkage map and QTL analysis have remained elusive until now.

Cytogenetic research shows that noug is a diploid species (2n = 2x = 30) with a comparatively small genome size, estimated to be around 1.5–1.7 Gb (Zhang et al., 2018). Its close relative is sunflower (*Helianthus annuus*), but it exhibits many differences from sunflower, where it is believed to have had a more complex evolutionary history, involving partial polyploidization (Badouin et al., 2017). In the face of this phylogenetic relationship and ecological and socio-economic value, noug is still one of the least studied in its genus concerning genetics worldwide. The lack of basic genomic information, such as a dense genetic linkage map or identified quantitative trait loci (QTL), has resulted in a serious bottleneck in genetic improvement through modern breeding approaches such as marker-assisted selection (Zhang et al., 2018). This disparity is even more striking when compared to leading oilseeds, including soybean and sunflower, which have witnessed substantial changes due to genomic tools. Therefore, to facilitate targeted breeding, this study aimed to (i) develop the first high-density SNP-based genetic linkage map for noug, and (ii) identify QTLs controlling key agronomic traits.

## 2 Materials and methods

### 2.1 Plant material

The initial germplasm was sourced from the Ethiopian Biodiversity Institute (EBI). This study used two groups of noug genotypes (Group-1 and Group-2). Group-1 consisted of 96 genotypes, including parents and their F_1_ progenies, used for parental selection. Two genetically distinct parents (Parent-1 and Parent-2) were selected from Group-1 to develop the F_2_ mapping population (Group-2), consisting of 286 progenies.

### 2.2 Greenhouse conditions and plant growth

All experiments were conducted in a greenhouse at the Swedish University of Agricultural Sciences (SLU), Alnarp, under controlled environmental conditions mimicking Ethiopia’s highland agroecology, with 16 hours of daylight at temperatures of 25°C/day and 21°C/night along with 60% relative humidity and 500 μmol/m²/s light intensity. Plants were grown in 2.5 L plastic pots containing standardized potting soil.

### 2.3 Leaf tissue sampling and DNA extraction

Leaf tissue was collected from two-week-old seedlings of Group-1 and Group-2 genotypes for DNA extraction and genotyping. Samples were obtained using the BioArk Leaf Collection Kit (LGC Biosearch Technologies) by punching ten 6-mm diameter discs from each plant, which were then placed in 96-well sampling plates. The collected tissue was shipped to LGC Biosearch Technologies (Berlin, Germany) for processing. High-quality genomic DNA was extracted using the Sbeadex Plant Kit (LGC Biosearch Technologies) and subsequently used for SeqSNP and genotyping-by-sequencing (GBS) analysis.

### 2.4 Selfing and crossing of group-1 genotypes

Twenty-one self-compatible genotypes derived from earlier breeding efforts (Geleta and Bryngelsson, 2010) were grown in the greenhouse for selfing and crossing. For self-pollination, individual branches were bagged before flowering. Cross-pollination was conducted by manually transferring pollen from donor flowers at 50% anther dehiscence to receptive stigmas of intact recipient flowers, followed by immediate bagging to prevent pollen contamination. Since recipient plants were self-compatible and not emasculated, the resulting seeds represent a mixture of selfed and hybrid progeny. At plant maturity, 21 seeds from self-pollinated capitula (one seed per plant) and 75 seeds from cross-pollinated capitula (3–4 seeds per plant) were sampled. These 96 seeds, representing the complete set of Group-1 genotypes, were subsequently planted in the greenhouse for genotyping.

### 2.5 Group-1 genotype sequencing for parental selection and F_1_ hybrid identification

Group-1 genotypes were genotyped using SeqSNP, a targeted genotyping-by-sequencing method (Geleta et al., 2024; Osterman et al., 2021). The SeqSNP assay targeted 300 bi-allelic SNPs derived from 300 of the 628 noug contigs published by Tsehay et al. (2020). Of these, 263 SNPs (most covered by two oligo probes) met high-specificity design criteria (no off-target hits permitted) and were selected for analysis. A SeqSNP kit containing 526 high-specificity oligo probes (two per SNP) was synthesized, and sequencing libraries were prepared. Target SNPs were sequenced on an Illumina NextSeq 500/550 v2 platform in 75-bp single-read mode. Sequencing yielded approximately 63,000 reads per sample on average, with an average effective target SNP coverage of 164×.

SNP calling, genotype assignment, and data filtering were conducted as described in Osterman et al. (2021). From this analysis, 145 high-quality polymorphic loci were selected for genetic characterization of the 96 genotypes, including assessments of genetic distance between the genotypes and identification of selfed progeny and F_1_ hybrids. From Group-1, we selected two genetically distinct parental lines (Parent-1 and Parent-2) based on their contrasting phenotypes for key traits: days to flowering (DTF), oil content (OC), and fatty acid composition (Geleta and Ortiz, 2013; Gebeyehu et al., 2021; Tsehay et al., 2021). Parent-1 displayed taller stature, later maturity, higher oil content, and lower oleic acid levels compared to Parent-2. These significant phenotypic differences, along with their genetic divergence, made them suitable for linkage analysis and QTL mapping.

We identified F_1_ hybrids by detecting heterozygous alleles at loci where the parental lines showed homozygous differences. The F_2_ mapping population was then developed through self-pollination of a single F_1_ hybrid derived from crossing Parent-1 and Parent-2.

### 2.6 Group-2 genotype phenotyping

Group-2 genotypes, consisting of 286 F_2_ progeny and the two parental lines, were phenotyped for nine phenotypic traits: plant height (PH, cm), number of seeds per plant (NSPP), number of capitula per plant (NCPP), capitulum size (CS, cm), flower size (FS, cm), days to flowering (DTF), thousand seed weight (TSW, g), oil content (OC, %), and oleic acid content (OAC, % of total fatty acids) (**Table 1**, **Supplementary Figure S1**). To ensure self-pollination, flowers were bagged pre-anthesis. PH, FS, and CS were measured in centimeters, while NCPP, NSPP, and DTF were recorded as counts. At maturity, seeds were harvested for TSW determination and subsequent gas chromatography (GC)-based analysis of OC and OAC, following the protocol described in Gebeyehu et al. (2024).

**Table 1:** Description of the characteristics investigated in this study.

Trait	Measurement	Mean ± SE in the F_2_ population	SD	Heritability (H²)
NCPP	Count	18.36 ± 0.73	9.31	0.41
NSPP	Count	18.64 ± 0.67	8.56	0.38
TSW	Gram	4.52 ± 0.05	0.60	0.67
OC	Percent	43.29 ± 0.94	11.95	0.72
OAC	Percent	31.86 ± 1.26	16.07	0.58
DTF	Count	84.00 ± 1.40	17.87	0.82
PH	Centimeter	208.31 ± 2.72	34.79	0.47
FS	Categorical	4.00 ± 0.05	0.59	0.63
CS	Categorical	3.18 ± 0.02	0.26	0.55

SD, standard deviation; SE, standard error; Categorical measurement: 1 = (small, < 3cm); 3 = (medium, 3–4 cm); 5 = (large, > 4cm).

### 2.7 Group-2 genotype sequencing

#### 2.7.1 Library construction, sequencing, and data pre-processing

For genetic linkage analysis and QTL mapping, Group-2 genotypes, comprising 286 F_2_ progeny and the two parental lines, were genotyped using genotyping-by-sequencing (GBS). To optimize library construction, multiple restriction enzymes were screened for fragment size distribution. Based on this evaluation, *PstI* and *ApekI* were selected for genomic DNA digestion, as they generated fragment sizes most suitable for GBS library preparation and sequencing. Constructed libraries were sequenced using Illumina NextSeq 500/550 v2 and NovaSeq 6000 FC platforms, generating 150 bp paired-end reads. The sequencing yielded approximately 288 million read pairs (one million per sample). Raw sequencing data were processed through base-calling and demultiplexing using Illumina’s bcl2fastq v2.20 software. Subsequent demultiplexing into individual samples was performed based on their inline barcodes and verification of the restriction site. Adapter remnants were clipped from all reads, and reads with a final length of <20 bases or lacking the expected restriction enzyme site at the 5′ end were discarded. Quality trimming included the removal of reads containing ambiguous bases (Ns) and 3′-end trimming using a 10-base sliding window with a minimum average Phred score of 20. Read quality metrics were assessed for all FASTQ files using FastQC v0.11.9.

#### 2.7.2 GBS clustering, alignment, variant discovery, and data filtering

Processed reads were clustered using CD-HIT-EST v4.6.1 (Li and Godzik, 2006), with a 5% sequence difference threshold. Singleton clusters and those with fewer than 20 reads were excluded. To ensure computational efficiency and minimize bias from uneven sequencing depth, reads were subsampled to a uniform depth of 1 million reads per sample using seqtk before alignment (Elshire et al., 2011). Subsampled quality-trimmed reads were aligned against the cluster reference using Bowtie2 v2.2.3, producing coordinate-sorted BAM files. Variant discovery and genotyping were performed using Freebayes v1.0.2–16 with stringent parameters, including a minimum base quality of 10, minimum coverage of 5, and ploidy of 2. Variants were filtered using a GBS-specific rule set: loci required a minimum read count of 8, genotypes had to be observed in at least 10% of samples, and the minimum allele frequency across all samples was set at 5%. Parental alleles were further filtered in relation to progeny.

The GBS analysis yielded a total of 294,818 cluster loci, with a high mapping rate of 90.6%. From these, 169,836 SNPs were identified across all samples, of which 85,457 loci were polymorphic. Applying a minimum read count threshold of eight and further filtering for SNPs with full coverage in at least 66% of the samples and a minor allele frequency of at least 5% resulted in a robust set of 13,888 high-quality SNPs. These markers were used for downstream genetic analysis. Sub-sampled quality-trimmed reads were also aligned to the sunflower reference genome (NCBI Assembly GCF_002127325.2) using BWA-MEM v0.7.12 (Li, 2013). Variant discovery and genotyping followed the same pipeline as used for the cluster reference alignment.

### 2.8 Linkage map construction using GBS-derived SNPs

The 169,836 GBS SNP markers were processed using VCFtools version v0.1.12a (Albers Cornelis et al., 2011) in the following order: (1) only SNPs with MAF of at least 40% were retained; (2) genotypes supported by a read depth of less than seven were set to missing; (3) SNPs with more than 10% missing data were discarded; (4) SNPs deviating from 1:2:1 segregation with p < 0.01 were discarded; (5) the SNPs were thinned so that no two SNPs were <65 bases apart (i.e., only one SNP was retained per 64-base GBS tag locus); and (6) the genotypes were converted to a numerical format to facilitate further SNP processing. Missing data (<10%) and MAF (>0.05) filters were applied to ensure robust SNP calling, consistent with similar studies (Elshire et al., 2011). The LOD threshold (3.0) was chosen based on permutation tests (1,000 iterations) to control false positives and balance computational efficiency and statistical robustness (Broman et al., 2003). Although strict SNP filtering ensures the data quality, it might have left out QTL with lesser effects (minor-effect QTL) or rare alleles. The GBS data were generated and analyzed at LGC Genomics GmbH, Germany, and 13,888 biallelic SNPs were generated, of which 742 SNPs were mapped to 15 LGs and used for QTL analysis.

A genetic map was developed with 15 linkage groups (LGs) and further processed for QTL analysis among the phenotypic data set using in-house scripts to generate an input file in *.bip format. Analysis of parameters involved the Kosambi mapping function with a variable inclusion standard of *P < 0.001*, genome scanning at 1 cM intervals, and a logarithm of odds (LOD) threshold of *≥* 3.0. The genetic map spanned 742 SNPs distributed across 15 linkage groups (LGs), with a mean marker interval of 2.1 cM. LG8 and LG11 exhibited recombination hotspots. LG8 and LG11 exhibited recombination hotspots. However, the relatively low SNP density (~50 SNPs per linkage group) may explain the failure to detect QTL in LGs 6, 7, 14, and 15 (Hammenhag et al., 2020). Increasing the marker density with whole-genome sequencing or targeted SNP arrays would enhance QTL coverage and reduce gaps in the genetic map (Geleta et al., 2020). Although this density is similar to other GBS-based oilseed research (e.g., Zhang et al., 2018; Geleta et al., 2020; Hammenhag et al., 2020), minor QTL resolution may be reduced in regions with gaps >10 cM (e.g., LGs 6, 7, 14, and 15).

### 2.9 QTL analysis and candidate gene identification

Quantitative trait locus (QTL) mapping was conducted for the nine phenotyped traits using the Inclusive Composite Interval Mapping (ICIM) method in ICIM software v4.2 (Meng et al., 2015). The analysis was performed on an F_2_ mapping population consisting of 163 genotypes, where the other genotypes were dead in the greenhouse experiment (**Supplementary Table S1**). A significance threshold of LOD > 3.0 (with 1,000 permutations to minimize false positives), determined through 1,000 permutations, was applied to identify statistically significant QTL. LOD score distributions and permutation-based significance thresholds are provided in **Supplementary Table S2** to support QTL detection. QTL flanking regions (~150 kb) were analyzed based on sunflower’s LD decay (~100 kb) and gene density. Homologous regions were identified using Basic Local Alignment Search Tool (BLAST) analysis against the annotated sunflower genome in the NCBI databases, and candidate genes were prioritized by functional annotation (e.g., *GPT1* for lipid metabolism). This approach aligns with studies in soybean and rapeseed (Badouin et al., 2017), which were utilized to identify potential candidate genes located between two adjacent SNP markers flanking the QTL. The sunflower genome was selected for this analysis due to the absence of a noug reference genome assembly, its close phylogenetic relationship with noug, a well-annotated genome, making our comparative genomic analysis efficient (Badouin et al., 2017).

### 2.10 Phenotypic data analysis

Phenotypic data for nine traits were analyzed using Minitab^®^ version 22.1 (Minitab Inc.; **Supplementary Table S1**). Pearson correlation analysis between traits was conducted. Following QTL mapping, phenotypic means were compared among SNP genotypes flanking QTL regions. Analysis was performed using Minitab^®^ 22.1 statistical software (Minitab Inc., https://www.minitab.com/en-us/) at *P < 0.05*.

## 3 Results

### 3.1 Phenotypic data analysis

In this study, flower size (FS) and capitulum size (CS) were measured in centimeters but treated as categorical variables (1 = small, < 3cm; 3 = medium, 3 to 4cm; 5 = large, > 4cm), a standard breeding practice for such traits in noug phenotyping, whereas the other seven traits were recorded as quantitative variables (**Table 1**; **Supplementary Figure S1**). The mean flower size and capitulum size in the F_2_ population were 4.0cm and 3.2cm, respectively. Plant height (PH) ranged from 110 to 292cm (mean = 208.3cm), and days to flowering (DTF) ranged from 49 to 115 days (mean = 84.0). The mean number of capsules per plant (NCPP) and seeds per plant (NSPP) were 18.4 and 18.6, respectively (**Supplementary Table S1**). The mean thousand-seed weight (TSW) was 4.5g, and the mean percent oil content (OC) and oleic acid content (OAC, also known as 18:1) were 43.3% and 31.9%, respectively (**Supplementary Figure S2**). Most F_2_ plants (60.7%) had below-average NCPP, whereas the remaining plants (39.3%) had above-average NCPP.

The number of seeds per plant ranged from 3.4 to 45.9, with 55.2% of F_2_ plants below the mean (**Supplementary Table S1**). In terms of days to flowering, 51%, 23%, and 26% of plants in the F_2_ population were early (≤ 84 days), medium (85–99 days), and late (≥ 100 days) maturing types, respectively. The majority of plants had desirable traits, including large flower size (FS ≥ 4.0cm, 55.8%) and capsule size (CS ≥ 3.0cm, 87.7%). The oil content of 38.7%, 21.5%, and 39.8% of plants in the F_2_ population was low (≤ 44%), medium (45–50%), and high (≥ 51%), respectively (**Supplementary Table S1**). The oleic acid content (18:1) was low (≤32), medium (33-40), and high (≥40) in 55%, 8%, and 37% of plants in the F_2_ population, respectively (**Supplementary Table S1**, **Figure S2**). Thousand-seed weight (TSW) was ≤ 5.0g in 83% of plants, while 17% produced seeds with TSW of > 5g. F_2_ plants displayed significant differences in height, with 55.8% of plants measuring 209cm or more, 14.7% ranging from 191 to 208cm, and 29.5% measuring below 190cm. This plant height range (110 to 292cm) exceeds the typical noug plant height range (140 to 200cm) under field conditions (Gebeyehu et al., 2021), showing the influence of greenhouse conditions on noug plants. Given that the phenotypic data were collected under controlled greenhouse conditions, which may not fully reflect field performance, particularly for traits like plant height and oil content that are sensitive to environmental variation (Gebeyehu et al., 2021). While greenhouse conditions control noise, multi-environment trials are planned to validate QTL stability under field conditions and assess genotype-by-environment (G×E) interactions. Such trials would help distinguish stable QTL from environment-specific effects and provide insights into potential G×E interactions, a critical step before deploying molecular markers in large-scale breeding programs (Dempewolf et al., 2015).

The Pearson correlation analysis revealed highly significant (P *<* 0.001) positive correlations between OC and OAC (r = 0.58), NCPP and PH (r = 0.36), NSPP and CS (r = 0.25), and NSPP and PH (r = 0.196), while a significant negative correlation (r = -0.18) was observed between NSPP and TSW (**Table 2**). While the phenotypic data revealed extensive variability across traits, the next step involved uncovering the genetic basis of these differences through high-resolution linkage mapping and QTL analysis.

**Table 2:** Pearson correlation coefficients between the nine traits in the F_2_ mapping population: Number of capitula per plant (NCPP), number of seeds per plant (NSPP), thousand seed weight (TSW), oil content (OC, %), oleic acid content (OAC, %); days to flowering (DTF); plant height (PH, cm); flower size (FS); and capitulum size (CS).

	NCPP	NSPP	TSW	OC	OAC	DTF	PH	FS	CS
NSPP	0.019								
TSW	0.005	-0.175^*^							
OC	-0.034	0.046	0.129						
OAC	-0.004	-0.029	0.075	0.579^***^					
DTF	0.018	-0.109	0.029	-0.144	-0.000				
PH	0.357^***^	0.196^*^	0.176^*^	-0.115	-0.048	0.099			
FS	0.129	0.082	0.083	-0.097	-0.110	0.048	0.263		
CS	0.091	0.245^**^	0.034	-0.027	-0.095	-0.010	0.437	0.231	

***P < 0.001, **P < 0.01, *P < 0.05; no asterisk means not significant.

#### 3.1.1 Determining correspondence between the noug linkage groups and the sunflower chromosomes

The noug GBS reads were mapped to the sunflower reference genome, where 5,823 SNPs were mapped to the 17 sunflower chromosomes, and a mapping summary coverage of *Guizotia abyssinica* (noug) SNPs aligned to *Helianthus annuus* (sunflower) chromosomes is provided in **Table 3**. The distribution of high-quality SNP loci (<10% missing data) across sunflower chromosomes, including total mapped loci and alternate allele frequencies, as compared to the reference genome (**Table 4**). This approach offers valuable insights into the comparative genomics and evolutionary relationships between noug and sunflower, even in the absence of a noug reference genome.

**Table 3:** Mapping coverage of *Guizotia abyssinica* (noug) SNPs aligned to *Helianthus annuus* (sunflower) chromosomes.

Sunflower chromosome	Reference sequence	Consensus length	Total read count	Single reads	Reads in pairs	Average coverage	Reference length
1	CM007890.2	3397270	40262652	2645784	37616868	18.23	149502186
2	CM007891.2	3389559	32521645	1936633	30585012	11.45	174800439
3	CM007892.2	3940619	49377383	3603369	45774014	18.75	176490873
4	CM007893.2	4281761	52505114	3377844	49127270	18.07	208320189
5	CM007894.2	3676362	41279590	2330302	38949288	16.67	178169690
6	CM007895.2	3208655	46114096	3306202	42807894	22.11	148147350
7	CM007896.2	2990627	32983415	2059927	30923488	14.33	149542083
8	CM007897.2	3555775	41857026	2911654	38945372	16.67	167167940
9	CM007898.2	4460126	57064400	3555200	53509200	21.20	189665024
10	CM007899.2	3887066	45784171	2903597	42880574	17.59	181411567
11	CM007900.2	3988308	42963837	2172365	40791472	15.11	189830405
12	CM007901.2	3455371	41168647	3294789	37873858	16.81	163781230
13	CM007902.2	3975540	53223404	3632716	49590688	21.12	173487274
14	CM007903.2	3973995	52883738	3130386	49753352	23.02	173346949
15	CM007904.2	3871855	46030018	2843536	43186482	16.65	175671323
16	CM007905.2	4338846	52300857	3352729	48948128	17.44	206736614
17	CM007906.2	4133152	71272955	3124567	68148388	35.80	195042445

**Table 4:** Distribution of high-quality SNP loci (<10% missing data) across sunflower chromosomes, including total mapped loci and alternate allele frequencies compared to the reference genome.

Sunflower chromosome	Reference sequence	No. of SNP loci	100% AF	90–99% AF	<90% AF	The last group	% loci with 100% AF	% loci with >90% AF
1	CM007890.2	109	62	29	18	<53%	56.9	83.5
2	CM007891.2	67	37	23	7	<62%	55.2	89.6
3	CM007892.2	116	52	25	39	<62%	44.8	66.4
4	CM007893.2	105	59	20	26	<74%	56.2	75.2
5	CM007894.2	98	59	18	21	<76%	60.2	78.6
6	CM007895.2	106	58	23	25	<87%	54.7	76.4
7	CM007896.2	85	43	17	25	<81%	50.6	70.6
8	CM007897.2	64	36	12	16	<76%	56.3	75.0
9	CM007898.2	138	81	27	30	<90%	58.7	78.3
10	CM007899.2	99	42	26	31	<77%	42.4	68.7
11	CM007900.2	100	53	18	29	<82%	53.0	71.0
12	CM007901.2	85	49	22	14	<53%	57.6	83.5
13	CM007902.2	119	70	20	29	<85%	58.8	75.6
14	CM007903.2	137	72	29	36	<80%	52.6	73.7
15	CM007904.2	83	46	23	14	<73%	55.4	83.1
16	CM007905.2	136	55	32	49	<83%	40.4	64.0
17	CM007906.2	86	38	25	23	<87%	44.2	73.3

AF, Allele frequency of alternate allele.

### 3.2 QTL analysis

We developed the first high-density SNP-based linkage map for noug, a significant milestone in the genomic research of minor oilseed crops. The results of this study are consistent with previous studies in sunflower (*Helianthus annuus*), whereby Badouin et al. (2017) were able to demonstrate the utility of comparative genomics to describe the evolution of the genome in Asteraceae. Our synteny exploration revealed that 11 out of 15 noug LGs are highly homologous with sunflower chromosomes, notably LG4 clustering with sunflower chromosomes 4 and 17 (60% coverage). Such conservation is in line with findings in rapeseed (*Brassica napus*), whereby Zhao et al. (2016) described such syntenic relations among chromosomes of different Brassica species.

**Figure 1:**
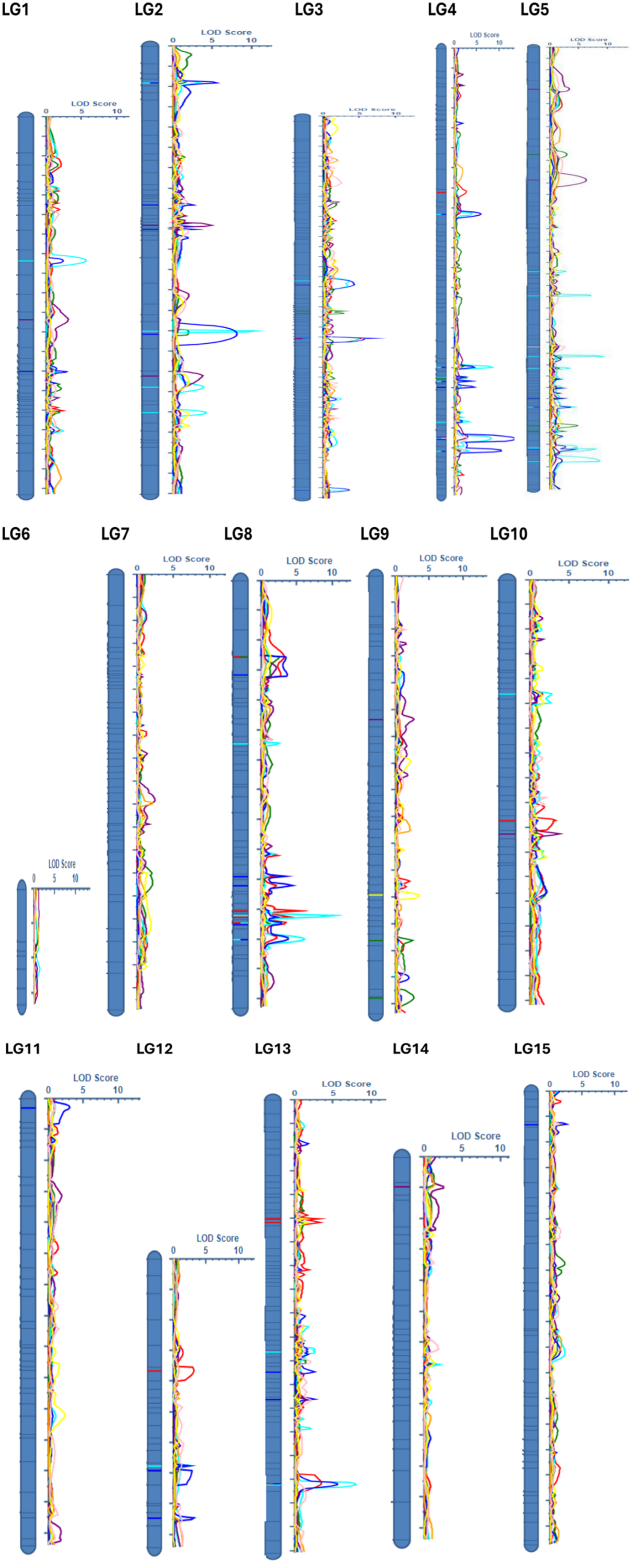
Distribution of the 15 quantitative trait loci (QTL) across *Guizotia abyssinica* linkage groups, showing LOD scores for six quantitative traits: number of capitula per plant (NCPP, red), number of seeds per plant (NSPP, green), thousand seed weight (TSW, turquoise), oil content (OC, purple), flower size (FS, blue), and days to flowering (DTF, yellow).

QTL were detected for 6 of the 9 noug traits evaluated (**Table 5**; **Supplementary Table S2**). These QTL were distributed across 11 of the 15 LGs, where none were detected for LG6, LG7, LG14, and LG15 (**Figure 1**). Phenotypic variation explained (PVE) was reported individually for each QTL and summed for traits with multiple QTL as cumulative PVE to reflect cumulative genetic effects (**Table 5**). QTL for TSW were concentrated mostly on LG4 (7), where 7 LGs collectively explained 51.2% of the observed phenotypic variation (**Table 5**; **Supplementary Table S2**). Most of the variation in the trait FS was explained by LG4 (6), followed by LG8 (4) and LG3 (3), collectively accounting for 47.5% of the variation across the 8 LGs. Five QTL collectively explained 32.46% of the PVE, where 26% of the variation for this trait was explained by LG2 (2) and LG5 (2). For the trait NCPP, 5 LGs collectively explained 32.8% of the PVE, where 4 QTL on LG8 and 3 QTL on LG13 collectively accounted for 26.2% of the variation, with single QTL on LGs 3, 10, and 12 each (**Table 5**). QTL for flowering time (qDTF-9-1) explained 7.6% PVE, while seed yield QTL (qNSPP-5-1) explained 2.9% (**Table 5**; **Supplementary Table S2**). The low phenotypic variation explained by some QTL (e.g., qNSPP-5–1 at 2.9% PVE) suggests that our F_2_ population size (n = 286) and greenhouse-controlled conditions may have biased the detection toward major-effect loci. At the same time, minor-effect QTL or those sensitive to environmental interactions (e.g., PH, OAC) likely remained undetected. Permutation tests (1,000 iterations) minimized false positives, but the absence of QTL in ‘cold spots’ (LGs 6, 7, 14, 15) and for polygenic traits underscores the need for validation in larger or advanced populations (e.g., recombinant inbred lines, RILs) and multi-environment trials. Hence, the low phenotypic variation explained by some QTL (qDTF-9–1 and qNSPP-5-1) in this population warrants caution, as population structure or environmental effects may inflate estimates (Broman et al., 2003). Further validation in advanced generations or diverse environments is needed to confirm their stability, effect sizes, and breeding relevance.

**Table 5:** Major QTL identified in noug (*Guizotia abyssinica*) linkage groups (LGs).

QTL name	Noug LGs	Trait	Flanking markers	Position (cM)	QTL interval (cM)	PVE (%)	Sunflower homolog (chromosome)	Candidate gene identified	Putative function	Arabidopsis homolog (AGI number)	References
qDTF-9-1	LG9	DTF	TP7155 - TP4346	61.7	~58-65	7.6	HaChr9	*CLC-b*	Regulate ion homeostasis and abiotic stress tolerance.	AT1G30450	(Li et al., 2020; Pantoja, 2021)
qNSPP-5-1	LG5	NSPP	TP5200 - TP238	42.6	~40-45	2.9	HaChr15	*GPT1*	lipid metabolism, pollen maturation, and seed development.	AT5G54800	(Niewiadomski et al., 2005)
qTSW-4-2	LG4	TSW	TP374 - TP5505	55.3	~55-58	8.1	HaChr4/17	*TL15.2*	involved in photosynthesis and drought stress responses.	AT2G35410	(Xia et al., 2019; Pakzad et al., 2019)
qOC-10-1	LG10	OC	TP2190 - TP5886	38.9	~36-42	11.3	HaChr1	*KDH*	links lipid biosynthesis with amino acid catabolism.	AT5G65750	(Zhou et al., 2024)
qFS-8-1	LG8	FS	TP7884 - TP9763	72.4	~70-75	9.8	HaChr8	*MCM2*	DNA replication and genomic stability during seed development.	AT1G44900	(Torres-Arroyo et al., 2024)
qNCPP-13-1	LG13	NCPP	TP8685 - TP9746	67.2	~65-70	12.7	HaChr12	*NUA*	mRNA export and nuclear pore organization.	AT1G79280	(Xu et al., 2007)

PVE (%), Phenotypic variation explained by individual QTL; QTL interval (cM), Interval range around the peak position. Candidate genes identified through homology to sunflower (*Helianthus annuus*).

Having identified these key genomic regions related to agronomic traits, we next examined homologous sequences in the related species *Helianthus annuus* to pinpoint candidate genes underlying the observed phenotypic variation. There were no QTL detected on genomic ‘cold spots’ (LGs 6, 7, 14, and 15). In addition, no QTL were detected for the traits PH and OAC, which might be because of low SNP density (50 SNPs per LG) in ‘cold spots’ alongside specific structural divergences within lineages may have hindered QTL detection. Targeted SNP arrays or whole-genome sequencing (WGS) could improve the coverage (Zhang et al., 2018). QTL detection power was evaluated through simulated thresholds, achieving ~80% power for QTL explaining ≥10% variance (LOD ≥ 3.0). With a trait heritability of 0.3–0.5, our design achieved approximately 80% power to identify QTL accounting for at least 10% of the phenotypic variance (LOD ≥ 3.0), while demonstrating reduced sensitivity for QTL contributing less than 5%. This is consistent with the lack of identified QTL for polygenic traits such as PH and OAC, which probably involve QTL with minor effects. Such cold spots are also likely caused by structural divergence from sunflower.

### 3.3 Comparative analysis of *G. abyssinica* linkage groups and *H. annuus* chromosomes

In this study, noug sequences were compared to *Helianthus annuus*, a close relative with a well-annotated genome, to identify homologous regions. The DNA sequence within each pair of flanking SNPs associated with QTL was BLAST searched against the *Helianthus annuus* genome in the NCBI database. For example, qDTF-9-1 (7.6% PVE) was linked to *CLC-b* after excluding 12 other genes in the 150 kb region with no known flowering-time function. QTL were identified for traits NCPP, NSPP, TSW, FS, DTF, and OC, but not for PH, OAC, and CS (**Figure 1**; **Supplementary Figure S3**), perhaps due to polygenic control, lack of marker coverage, or lowered phenotypic variation under greenhouse conditions (Hammenhag et al., 2020). An F_2_ population size and greenhouse conditions may be biased towards major-effect loci during QTL identification. Genomic selection or GWAS using multiple landraces would overcome these limitations (Gupta et al., 2017; Zhang et al., 2018). Of the 15 LGs, 11 LGs had multiple hits with the *H. annuus* chromosomes, but to varying extents (**Table 6**). However, four LGs (LG6, 7, 14, and 15) do not have homologous regions in the *H. annuus* genome (**Table 6**, **Figure 1**). LG4 showed the strongest synteny with *H. annuus* chromosomes 4 and 17 (60% coverage), a region harboring QTL associated with thousand-seed weight (TSW). A total of 8,580 bp matching sequences were found for LG4, where 5,451 bp, 1,642 bp, and 1,487 bp sequences were matching sequences with *HaX-4, HaX-6*, and *HaX-17*, respectively (**Table 6**). Moreover, the largest groups of matching sequences for the trait OC were from *HaX-6*, followed by *HaX-11* and *HaX-7* at LG4 (3,070 bp), LG10 (1,770 bp), and LG2 (1,544 bp), respectively. FS was shared among LGs 2, 4, 5, 8, 11, and 12, and the largest group of matching sequences were at LGs 4 and 8 (3,070 bp each), followed by LG11 (2,372 bp), where these sequences were matching sequences with *HaX-6* and *HaX-8*, respectively (**Table 6**; **Supplementary Table S2**). The number of capitulum per plant (NCPP) was shared among LGs 3, 10, 12, and 13, and the largest group of matching sequences were at LGs 12 and 13 (6,638 bp each), followed by LG10 (2,372 bp), where these sequences were matching sequences with *HaX-12* and *HaX-1*, respectively. In general, LGs 3, 12, and 13 shared 100% sequence identity with *H. annuus* chromosome 12 for the trait NCPP (**Table 6**; **Supplementary Table S2**). The traits DTF and NSPP shared 100% sequence identity with *H. annuus* chromosomes 9 and 12, respectively (**Table 6**; **Supplementary Table S2**).

**Table 6:** Assembled size of 11 of the 15 *G. abyssinica* linkage groups (LGs) and their homology to *H. annuus* chromosome (*HaX*): Sequence identity, alignment length, number of identified noug QTL, and corresponding chromosomal regions.

Linkage group (LG)	Sequence size (bp)	*H. annuus* chromosome (*HaX*)	Total score	Number of QTL found in LGs	Number of matches with *H. annuus*	Noug traits detected	*H. annuus* accession number (Sequence ID)
LG1	829	13	1531	1	0	TSW	XM_022145194.2
LG2	6638	13	12259	4	1	TSW	XM_022145194.2
	829	13	1531	1	1	FS	XM_022145194.2
	1544	7	2852	2	1	OC	XM_022137020.2
LG3	4022	12	7428	1	3	NCPP	XM_022143038.2
LG4	5451	4	1006	6	4	TSW	XM_035989027.1
	1642	6	3033				XM_022114098.2
	1544 1487	17 17	2852 2747				XM_022137020.2 XM_022137021.2
	3070	6	5670	6	3	FS	XM_022114430.2
	3070	6	5670	1	1	OC	XM_022114430.2
LG5	1674	15	3092	1	1	NSPP	XM_035983992.1
	1338	7	2471	7	3	TSW	XM_022127051.2
	1338	7	2471	2	1	FS	XM_022127051.2
LG8	3070	6	5670	4	1	FS	XM_022114430.2
LG9	2771	9	5118	1	1	DTF	XM_022126734.2
LG10	4386	1	8100	1	1	NCPP	XM_022122457.2
	1770	11	3269	1	1	OC	XM_022123420.2
LG11	2372	8	4381	1	1	FS	XM_022121845.2
LG12	6638	12	12259	1	1	NCPP	XM_022150738.2
	1772	11	3273	1	1	FS	XM_035979698.1
LG13	6638	12	12259	3	1	NCPP	XM_022143038.2

*H. annuus* accession numbers (sequence ID) refer to *H. annuus* sequences in the NCBI database. The noug Linkage Group (LG) sequences had a mean G+C content of 35%, 100% query coverage and sequence identity with *H. annuus* sequences, and zero E-values (0.0) for all alignments. PVE—phenotypic variation explained by individual QTL.

**Figure 2:**
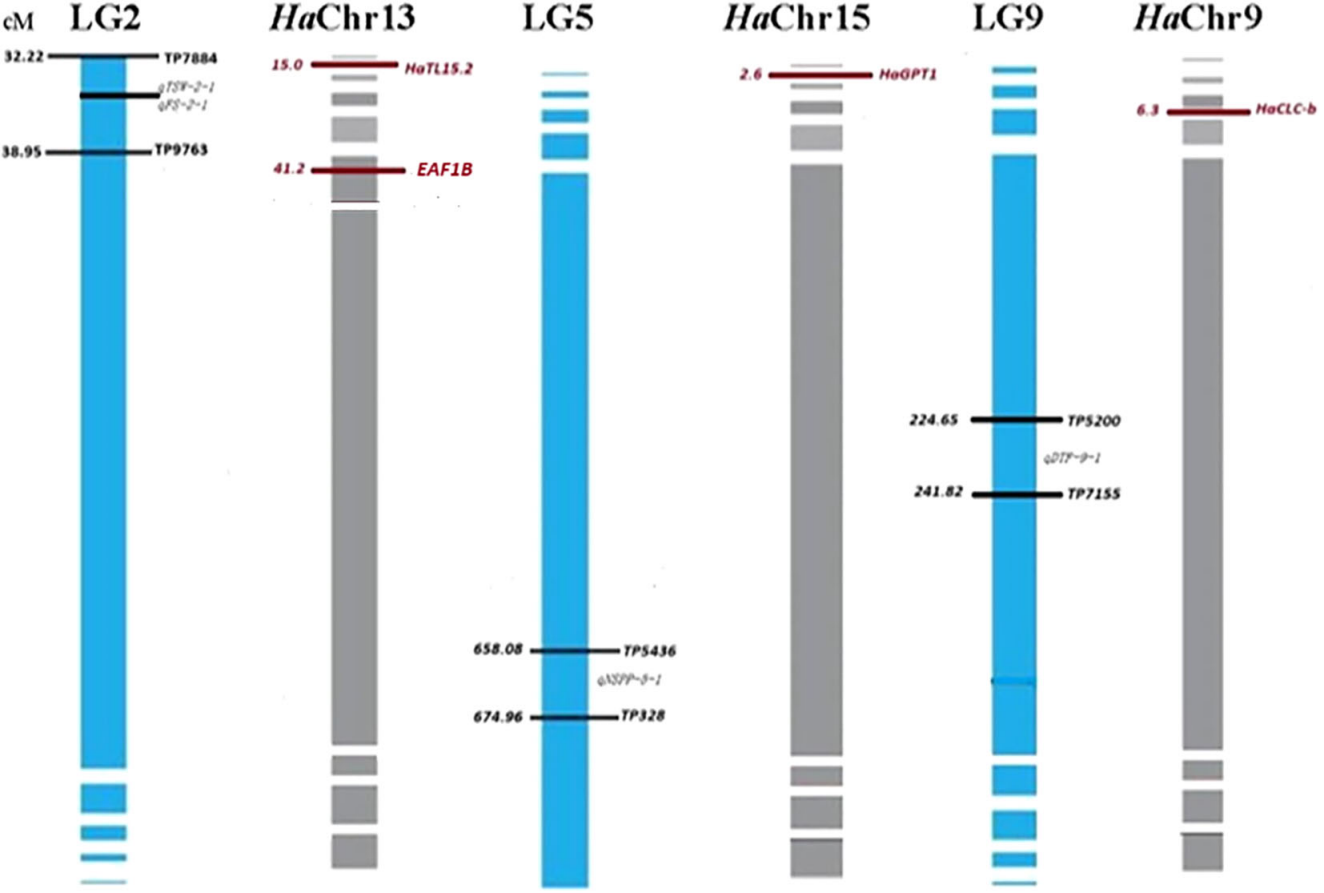
Candidate gene identification through targeted synteny analysis of noug (*Guizotia abyssinica*) with sunflower (*Helianthus annuus*). The figure illustrates the comparative genomics approach used to identify candidate genes underlying key QTLs by leveraging homologous regions between *Guizotia abyssinica* linkage groups and *Helianthus annuus* chromosomes. QTL positions (cM) for seed weight (qTSW-2-1), flower size (qFS-2-1), number of seeds per plant (qNSPP-5-1), and flowering time (qDTF-9-1) are mapped to sunflower chromosomes (*Ha*Chr9, *Ha*Chr13, and *HaChr15*)*. H. annuus* homologs are shown in red.

Analysis of the homologous regions (synteny) between the flanking markers from *G. abyssinica* LGs and *H. annuus* chromosomes (*HaChr9, HaChr13*, and *HaChr15*) was performed, and candidate genes for QTL controlling NSPP, TSW, FS, and DTF were detected (**Figure 2**). The *G. abyssinica* candidate gene qTSW-2–1 at LG2 was homologous to *H. annuus TL15.2* (chromosome 13), a gene involved in photosynthesis. In addition, the EAF1B protein regulates plant developmental processes and the transcriptional activation of specific genes (Parakkunnel et al., 2022; Wang et al., 2019) and is homologous to the candidate gene qFS-2–1 at LG2. Hence, both qTSW-2–1 and qFS-2–1 at LG2 are likely candidate genes for flowering and seed setting in noug. The *G. abyssinica* candidate gene qNSPP-5–1 at LG5 was homologous to the *H. annuus GPT1* gene at chromosome 15 (**Figure 2**), which regulates lipid metabolism and seed development in sunflower. Furthermore, the candidate gene qDTF-9–1 at LG5 was homologous to the *H. annuus CLC-b*, which regulates flowering time. In summary, LG4 showed 60% synteny with sunflower chromosomes 4 and 17, LG5 aligned with HaChr15 (*GPT1*), while LG9 aligned with HaChr9 (*CLC-b*). While candidate genes (*CLC-b, GPT1*, and *TL15.2*) were identified based on homology to sunflower, future functional validation using transcriptomics could help confirm gene-trait relationships in noug.

## 4 Discussion

A genetic linkage map construction is the fundamental step in identifying genes and associated molecular markers for plant breeding. These findings highlight the potential of integrating genomic tools or transcriptomics for functional validation in noug. Previous studies have identified QTL related to seed yield and oil quality in oilseed plants, including *Brassica napus* (Geng et al., 2016; Zhao et al., 2016) and *Lepidium campestre* (Zhang et al., 2018; Geleta et al., 2020; Hammenhag et al., 2020), and yet the genetic mechanisms for these traits in noug remain largely unknown.

The gene composition preserved between noug LG9 (qDTF-9-1) and sunflower chromosome 9 (*CLC-b*) is comparable to what has been reported in other oil crops. In soybean (*Glycine max*), for instance, Qu et al. (2017) found conserved flowering time QTL in closely related legume species. Similarly, the lipid metabolism gene *GPT1* on noug LG5 is homologous with sunflower chromosome 15, as in rapeseed, where conserved oil biosynthesis genes were found in Brassica species (Geng et al., 2016). However, four noug LGs (6, 7, 14, and 15) showed no synteny with sunflower chromosomes, likely due to species-specific rearrangements, where similar patterns were reported in flax (*Linum usitatissimum*) sunflower homologs, similar to species-specific chromosome rearrangements reported in flax by Zhang et al. (2018). The structural disparities can be one explanation for the failure to detect QTL in these regions, i.e., “cold spots” in *Lepidium campestre* by Hammenhag et al. (2020). The relatively narrow genetic distance of LG2, despite widespread QTL parallel discoveries in sunflower by Ma et al. (2022), reported recombination suppression near centromeres.

The relationship between genetic and physical distances in noug presents interesting comparisons with other oil crops. Although our study lacked physical length estimates, the sunflower’s genome (∼3.5 Gb) has ∼1.6-2.2 cM/Mb (Badouin et al., 2017), suggesting that noug’s smaller genome (∼1.7 Gb) may have a higher recombination density. This contrasts with rapeseed’s ∼0.7 cM/Mb (Zhao et al., 2016), showing varying recombination landscapes in oil crops. Both low genetic distances and high marker density in LG8 and LG11 are analogous to recombination hotspots (Gupta et al., 2017).

The candidate genes *CLC-b, GPT1*, and *TL15.2* are functionally conserved across oilseed crops. The role of the *CLC-b* chloride channel protein in flower time regulation is complemented by research in sunflower by Li et al. (2020), and the involvement of *GPT1* in lipid metabolism agrees with Niewiadomski et al.’s (2005) studies in Arabidopsis. The role of the thylakoid lumen protein *TL15.2* in drought responses is verified by Xia et al.’s (2019) studies in industrial hemp.

These genomic resources pave the way for marker-assisted breeding in noug, corresponding approaches already used in crops like rapeseed and sunflower (Dempewolf et al., 2015) and rapeseed (Qu et al., 2017; Zhang et al., 2018; Hammenhag et al., 2020). However, according to Geleta et al.’s (2020) study in Lepidium, under-resourced crops require additional tools, e.g., a completed reference genome assembly, physical mapping via FISH/Hi-C, high-density SNP arrays for gaps in LGs 6, 7, 14, and 15, and multi-environment QTL validation. Hence, multi-parent populations or genomic selection could be implemented to dissect complex traits like plant height and oleic acid content. Future genetic maps should integrate chromosome-scale assemblies and multi-parent populations to resolve QTL “cold spots.” Combining WGS-level SNP density with haplotype-based QTL models could uncover minor-effect loci masked in this study, particularly for polygenic traits like PH and OAC.

### 4.1 Identification of significant QTL for tested traits

QTL were annotated to 11 of the 15 LGs, which is consistent with previously reported haploid chromosome numbers (15) for *G. abyssinica* (Dagne and Jonsson, 1997). These QTL collectively explained substantial portions of the phenotypic variation for the traits analyzed, with traits exhibiting polygenic architectures (**Supplementary Table S2**). Stringent LOD thresholds (3.0) and the F_2_ population size (n=286) likely limited the detection of minor-effect QTL (<5% variance). Nonetheless, QTL were identified for days to flowering (qDTF-9-1, 7.6% variance) and seed yield (qNSPP-5-1, homologous to sunflower *GPT1*). Although some QTL identified explain relatively small proportions of phenotypic variation (e.g., qNSPP-5-1, 2.93% variance), these may represent minor-effect loci that contribute to trait stability under variable environmental conditions. Their inclusion in breeding programs through genomic selection or pyramiding strategies could help improve complex traits incrementally. The remaining QTL accounted for 11.3–38.1% of the variation in oil content, flower size, and capitulum size, with significant correlations (r = 0.579) between oil content and oleic acid content.

The flowering time (qDTF-9-1) and seed yield (qNSPP-5-1) QTL showed divergent heritability patterns. Days to flowering was highly heritable, while the number of seeds per plant exhibited moderate heritability (H² = 31.6%) (Gebeyehu et al., 2021). The negative correlation between DTF and NSPP (*P* < 0.01) suggests environmental influences on yield, with no genetic trade-off. The independent inheritance of qDTF-9–1 and qNSPP-5–1 enables breeding for early maturity and high yield, critical for Ethiopia’s short growing seasons. These loci’s independent inheritance pattern indicates how noug’s domestication history stands apart or how agroecological niche pressures created distinct selection forces (Dempewolf et al., 2015). However, more studies would be needed to confirm this independence using field trials and determine if epistatic interactions under stress become evident.

Notably, the greenhouse environment, while reducing noise, may have constrained phenotypic variation for traits like plant height (which exceeded field-typical ranges) and oil composition, further limiting QTL detection. This bias toward major-effect loci is common in F_2_ populations (Broman et al., 2003), and our results align with similar studies in under-resourced crops (Zhang et al., 2018; Geleta et al., 2020; Hammenhag et al., 2020). Our F_2_ mapping population size was suitable for detecting major-effect QTL, but may lack statistical power for identifying minor-effect QTL (Broman et al., 2003). The absence of PH and OAC QTL may reflect polygenic control or undetected epistasis, where interactions between minor-effect loci (e.g., *FAD2* homologs) could collectively shape traits. Future studies should test epistatic models in expanded populations or diverse environments. Future studies could employ larger populations or advanced generations (e.g., RILs) to improve resolution (Zhang et al., 2018). Plant height showed small genotypic variation (GCV < 1%), with environmental variance (σ^2^_e_) hiding genetic influences (Gebeyehu et al., 2021). For low-heritability characters, larger sample sizes are recommended to increase the statistical power of the experiment and better estimate the genetic component of trait variation (Broman et al., 2003). Moreover, greenhouse conditions may have restricted the observed phenotypic variation, where only 31.9% of the mean OAC was explained in the present study, and the effect of temperature on OC and OAC (Gebeyehu et al., 2024). While our marker density (2.1 cM average interval) and population size (286) align with prior QTL studies in *Brassica napus* (Zhao et al., 2016) and *Lepidium campestre* (Hammenhag et al., 2020), polygenic traits may require genomic selection to capture minor-effect QTL, GWAS in diverse landraces to exploit historical recombination, or multi-environment trials to dissect G×E interactions obscured by greenhouse conditions. Functional validation of candidate genes (e.g., *CLC-b* and *GPT1*) using CRISPR-Cas or transcriptomics is needed to confirm their roles in noug.

### 4.2 Trait-based candidate gene analysis

#### 4.2.1 Days to flowering

Early-maturing crops need to be developed in Ethiopia because of the short growing season, underscoring the importance of releasing early-maturing cultivars. A QTL on LG9 was identified that accounts for 7.6% of the observed variation in DTF, with 26% of F_2_ plants exhibiting late maturity and 51% exhibiting early maturity (< 84 days). The negative association between yield and late maturity indicates strong G×E interactions. The *CLC-b* gene was identified in the noug LG9, which is homologous to the *H. annuus* chromosome 9. It codes for a chloride channel protein that is involved in abiotic stress tolerance (Pantoja, 2021), ion transport-related photosynthetic activity of chloroplasts, and salinity-controlled ion homeostasis (Li et al., 2020). The association of the *qDTF-9–1* locus with the *CLC-b* gene suggests it may be a useful target for selecting early maturity in noug. Even though the regulation mechanism of the *CLC-b* gene is still unknown in noug, it is an essential candidate gene for MAS to achieve early-maturing lines under Ethiopian short growing seasons.

#### 4.2.2 Number of capitulum per plant

The F_2_ mapping population showed variation in NCPP, with low counts for many plants. The genetic sequences on LG3, LG12, and LG13 aligned completely with *H. annuus* chromosome 12, whereas LG10 matched chromosome 1 sequences, while LG8 showed no matches for this trait. The four QTL qNCPP-3-1, qNCPP-10-1, qNCPP-12-1, and qNCPP-13-1, which mapped to LG8 and LG13, explained 70% of the observed phenotypic variation. About 60.7% of the F_2_ plants exhibited low capitulum counts, highlighting the polygenic regulation of the trait. The genes detected on LG3 for this trait were homologous to the *H. annuus* DExH3, which is responsible for RNA metabolism processes for abiotic stress tolerance, such as making ribosomes and digesting pre-ribosomal RNA (Liu and Imai, 2018). The candidate gene on LG10 is a homolog of the *H. annuus* disease-resistance protein *At*4g27190, which is part of the NBS-LRR family and is known for helping plants fight off infections (Neupane et al., 2018; Ma et al., 2022). The *H. annuus* Nuclear-Pore Anchor (NUA) protein, which facilitates the movement of mRNA and the organization of nuclear pores (Xu et al., 2007), is homologous to the noug LG12 candidate gene. Furthermore, functional similarity between LG13 and LG3 reveals similar functions in stress response and RNA metabolism in noug. This study, therefore, suggests that candidate genes underlying NCPP may be involved in developmental and stress-response pathways.

#### 4.2.3 Number of seeds per plant

The number of seeds per plant (NSPP) is a key yield component influenced by genetic and environmental interactions. The majority (55.2%) of the plants in the F_2_ mapping population had lower NSPP, while 44.8% had large seed counts (**Supplementary Table S1**). The low NSPP count in most F_2_ populations corresponds with expected genetic bottlenecks throughout the noug domestication process (Dempewolf et al., 2015). Seed number per plant had a positive correlation with PH (r = 0.196) and CS (r = 0.245) and a negative correlation with TSW (r = -0.175). This negative correlation reflects a trade-off in resource allocation to seed growth, where limitations on photosynthetic assimilates are divided between more and smaller seeds and fewer but larger seeds. This finding aligns with earlier research on sunflower and rapeseed, which shows that the average weight of seeds produced by a plant tends to decrease as the NSPP increases, and vice versa (Dempewolf et al., 2015; Zheng et al., 2018). The qNSPP-5–1 QTL explained low PVE (2.93%), indicating the need for validation in larger populations and varied environments. However, its homology to sunflower *GPT1*, a gene governing lipid metabolism and seed development (Niewiadomski et al., 2005; Liu et al., 2022; Zhou et al., 2024), and the observed trade-off between NSPP and TSW suggests potential biological relevance. Although unvalidated in noug, *GPT1’s* conserved role in sunflower supports its candidacy for increasing oilseed yield without trade-offs with other agronomic characters. This gene is also involved in pollen development, seed filling, and maturation (Zheng et al., 2018), as well as stress adaptation using protein acylation-mediated responses (Sharma et al., 2023). Hence, validation in larger populations or under field conditions is needed to confirm its utility for breeding.

#### 4.2.4 Thousand-seed weight

Thousand-seed weight (TSW) is an essential agronomic characteristic that determines the seed quality and germination potential, as it serves as the nutrient reserve during seedling establishment. Significant positive correlation with PH (r = 0.176) and significant negative correlation with NSPP (r = -0.175) were found. These negative correlations among yield traits suggest genetic and environmental trade-offs, which are consistent with previous research (Dempewolf et al., 2015; Zheng et al., 2018). The *H. annuus* thylakoid lumenal protein (*TL15.2*), which is linked to photosynthesis and drought stress responses (Xia et al., 2019; Pakzad et al., 2019), was found to be homologous to qTSW-1–1 and qTSW-2-1. Other potential genes from LG4 and LG5 that were homologous to the *H. annuus* transcription factor RAX2 and the chromatin modification-related protein *EAF1B* were detected, both of which are implicated in developmental processes and stress responses (Wang et al., 2019). Hence, favorable alleles at the TP8685 and TP9746 marker loci linked to *qTSW-1-1*, TP7884 and TP9763 linked to qTSW-2-1, TP374 and TP5505 linked to *qTSW-4-2*, and TP2190 and TP5886 linked to *qTSW-5–7* QTL may be useful for selecting noug plants with increased TSW.

#### 4.2.5 Flower size

Flower size is a crucial trait affecting pollination, seed set, and overall yield in noug. The flower size QTL present in LG2 and LG5 were homologous to qTSW-2–1 and qTSW-5-1, which may have a role in seed development and flower bud initiation. The flower size QTL at LG11 and LG12 were also homologous to the sunflower *MCM2.* Additional homologies were found with *the* sunflower HaNVL protein and HaWAK2 receptor kinase, involved in stress responses (Torres-Arroyo et al., 2024). The homology of these QTL to *MCM2* implies a possible relationship between DNA replication, genome stability, and the sunflower immune response (Bezuidenhout, 2006). Hence, marker-assisted selection using the flanking markers at *qFS-4-5*, *qFS-8-1*, *qFS-11-1*, and *qFS-12–1* can efficiently select noug plants with preferred flower size characteristics.

#### 4.2.6 Oil and oleic acid content

This research revealed that noug oil content varied extensively from 13.88% to 55.62%, with a mean value of 43.29%, and contained valuable unsaturated fatty acids such as oleic and linoleic acids (**Supplementary Table S1**). Previous research indicates that noug oil content varies between 42% and 44% (Dagne and Jonsson, 1997), and between 27% and 56% (Geleta et al., 2011), which aligns with our findings. Oleic acid content was reported to range from 5.4 to 27% (Dagne and Jonsson, 1997), 3.3 to 31% (Geleta et al., 2011), 23 to 53% (Yadav et al., 2012), 5.2 to 9.2% (Tsehay et al., 2021) under field conditions and 14 to 36% under greenhouse conditions at 21 °C to 25 °C (Gebeyehu et al., 2024), yet greenhouse conditions may not accurately reflect field conditions. The strong correlation between OC and OAC (r = 0.579) aligns with prior studies (Petros et al., 2009; Geleta et al., 2011; Tsehay et al., 2021). The correlation between OC and OAC might be explained by shared genetic control, even though we did not conduct a multi-trait QTL analysis. In future research, multivariate models will be employed to dissect these relationships.

Candidate gene screening identified three genomic regions that control oil-related traits in noug. LG2 (qOC-2-1) contains *Ha*CBP39, a calcium-binding protein associated with lipid metabolism (Murphy, 2020; Miklaszewska et al., 2021). The second locus (qOC-4-1), LG4, contains *MCM2*, a critical gene involved in DNA replication and genomic stability during seed development (Torres-Arroyo et al., 2024). Finally, LG10 contains the *qOC-10–1* locus, which is homologous to the *H. annuus KDH* gene, a gene that links lipid biosynthesis with amino acid catabolism (Zhou et al., 2024).

## 5 Conclusion

This study establishes the genetic basis of key agronomic traits through the construction of a high-density linkage map and identification of QTL for noug. Notably, qDTF-9-1, linked to the *CLC-b* gene, was implicated in having an influence on flowering time and stress acclimation, with a potential entry for breeding early maturity cultivars without compromising yield. Similarly, qNSPP-5-1, a *GPT1* homolog involved in lipid metabolism and seed development, has an immediate application for marker-assisted selection to improve seed yield. Furthermore, *TL15.2* (drought tolerance) and EAF1B (developmental regulation) QTL provide greater scope for targeted breeding. These QTL can guide the introgression of favorable alleles into elite lines. Future multi-environment trials will validate QTL stability and assess genotype-by-environment interactions to ensure breeding relevance. This study provides actionable markers (e.g., *CLC-b* for early flowering) and underscores the need for multi-environment trials to deploy these tools in breeding programs. However, homology-based hypotheses must be validated through transcriptomics, gene editing, and multi-location trials. Our findings support Ethiopian breeding programs with actionable markers and provide insight into the genetic regulation of key traits in noug. In conclusion, this work is a basis for precision breeding in the interest of food security, climate resilience, and agricultural sustainability in Ethiopia and similar climates.

## Data Availability

The raw sequencing data are deposited in the NCBI Sequence Read Archive (SRA) and are publicly accessible at BioProject PRJNA1193105 (accession number: SAMN45132013). PRJNA1193105 (*Guizotia abyssinica* cultivar: noug (ID 1193105) - BioProject - NCBI). Phenotypic data are available at **Supplementary Table S1**, and comparative analysis of *G. abyssinica* linkage groups (LGs) and their homology to *H. annuus* chromosome (*HaX*) is available at **Supplementary Table S2**.

## Supplementary material

Supplementary Table 1 List of the 163 F_2_ mapping population used in the present study, showing data collected for the nine quantitative (sheet 1) and three qualitative (sheet 2) characteristics.

Supplementary Table 2 Trait-based comparative analysis of *G. abyssinica* linkage groups (LGs) and their homology to *H. annuus* chromosome (*HaX*): phenotypic variation explained (PVE), interval length, position of identified noug QTL, and corresponding *H. annuus* chromosomal regions.

Supplementary Figure 1 Distribution of phenotypic data for the F_2_ mapping population. Histograms show the frequency distribution for plant height (PH), number of seeds per plant (NSPP), number of capitula per plant (NCPP), days to flowering (DTF), thousand seed weight (TSW), oil content (OC), and oleic acid content (OAC). A normal distribution curve is fitted to each histogram.

Supplementary Figure 2 Chromatographic depiction of the fatty acid profile of noug F_2_ mapping populations having low **(A)** and high **(B)** oleic acid (18:1) contents.

Supplementary Figure 3 Distribution of the 15 quantitative trait loci (QTL) across *Guizotia abyssinica* linkage groups for six quantitative characteristics: number of capitulum per plant (NCPP, red), number of seeds per plant (NSPP, green), thousand seed weight (TSW, turquoise), oil content (OC, purple), flower size (FS, blue), and days to flowering (DTF, yellow).
