# Supplementary figures and images for: SNP-based linkage mapping reveals novel quantitative trait loci for yield traits in noug (Guizotia abyssinica (L. f.) Cass.)
Source: Front Plant Sci. 2025 Sep 9;16:1662582. doi: 10.3389/fpls.2025.1662582 (PMC12454408; doi:10.3389/fpls.2025.1662582)

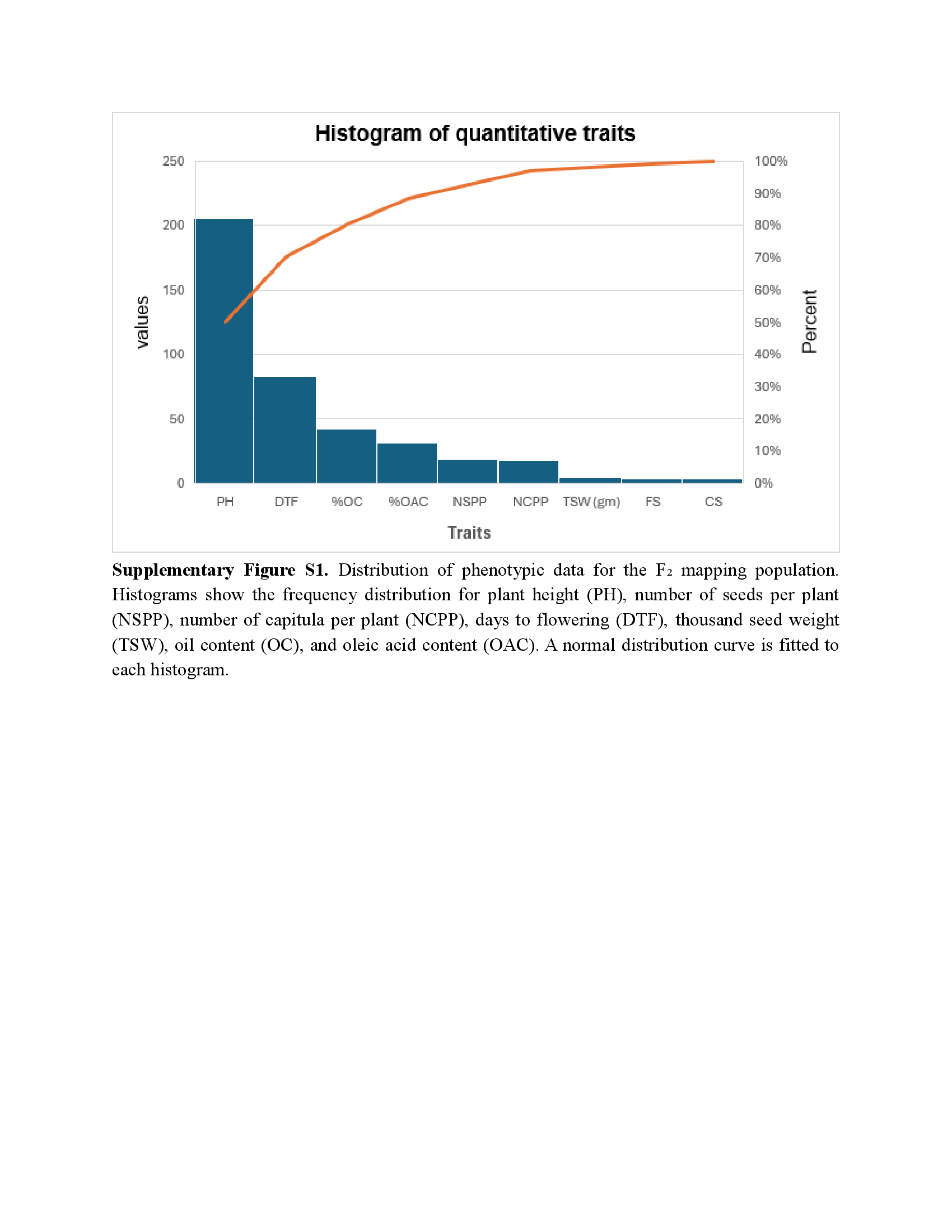

Supplement: Supplementary Figure 1 — Distribution of phenotypic data for the F2 mapping population. Histograms show the frequency distribution for plant height (PH), number of seeds per plant (NSPP), number of capitula per plant (NCPP), days to flowering (DTF), thousand seed weight (TSW), oil content (OC), and oleic acid content (OAC). A normal distribution curve is fitted to each histogram. [file Image1.tiff]

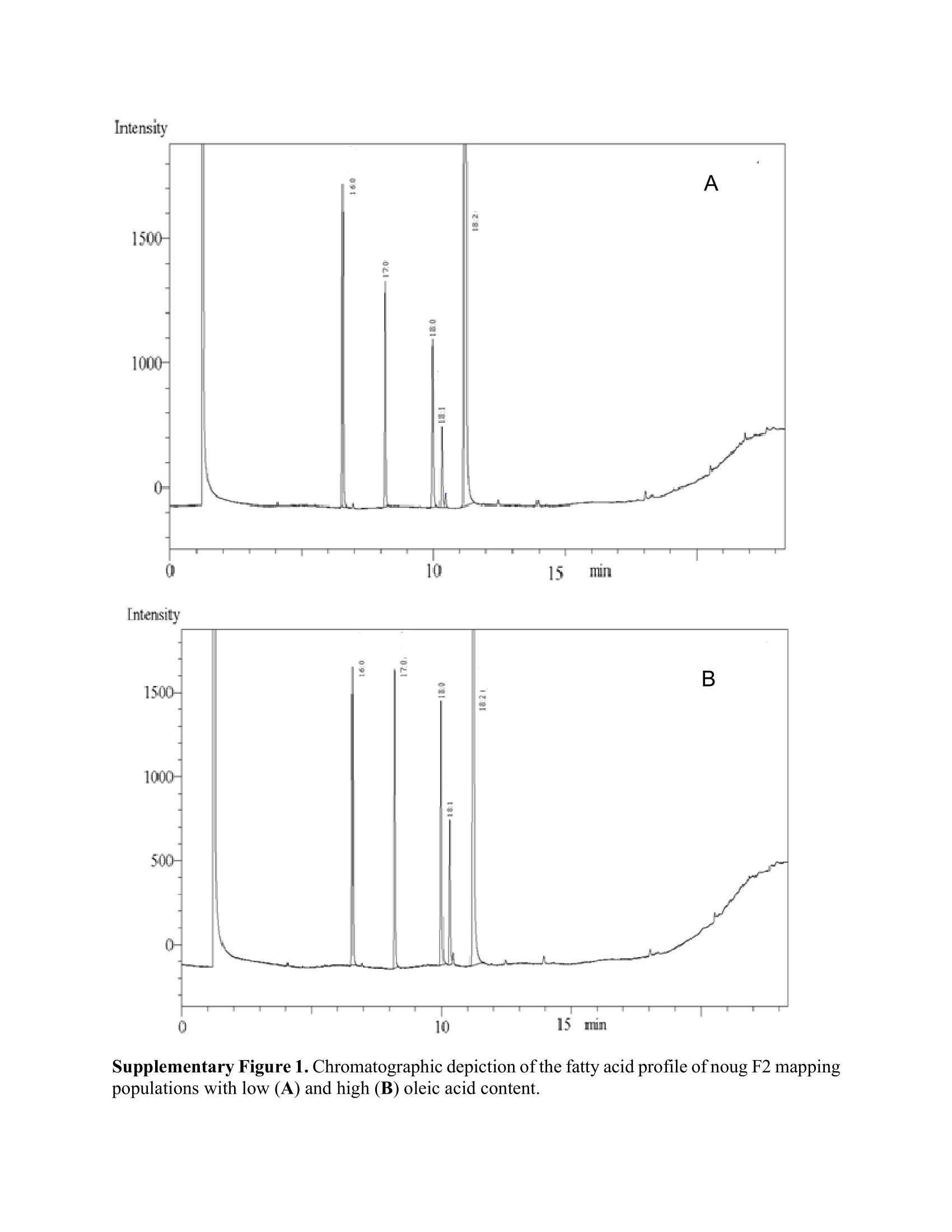

Supplement: Supplementary Figure 2 — Chromatographic depiction of the fatty acid profile of noug F2 mapping populations having low (A) and high (B) oleic acid (18:1) contents. [file Image2.tiff]

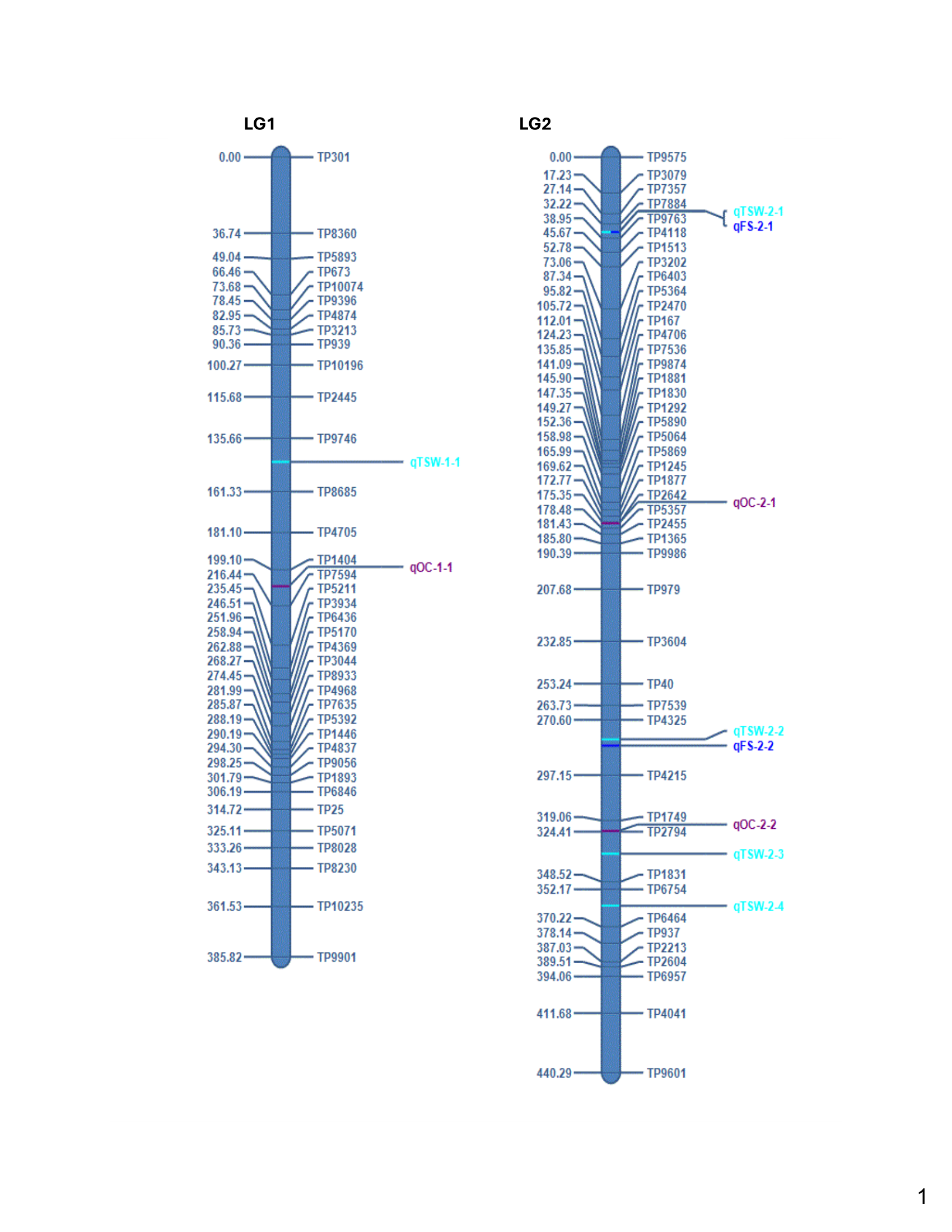

Supplement: Supplementary Figure 3 — Distribution of the 15 quantitative trait loci (QTL) across Guizotia abyssinica linkage groups for six quantitative characteristics: number of capitulum per plant (NCPP, red), number of seeds per plant (NSPP, green), thousand seed weight (TSW, turquoise), oil content (OC, purple), flower size (FS, blue), and days to flowering (DTF, yellow). [file DataSheet1.zip › Supplementary Figure S3/Supplementary Figure S3-1.tiff]

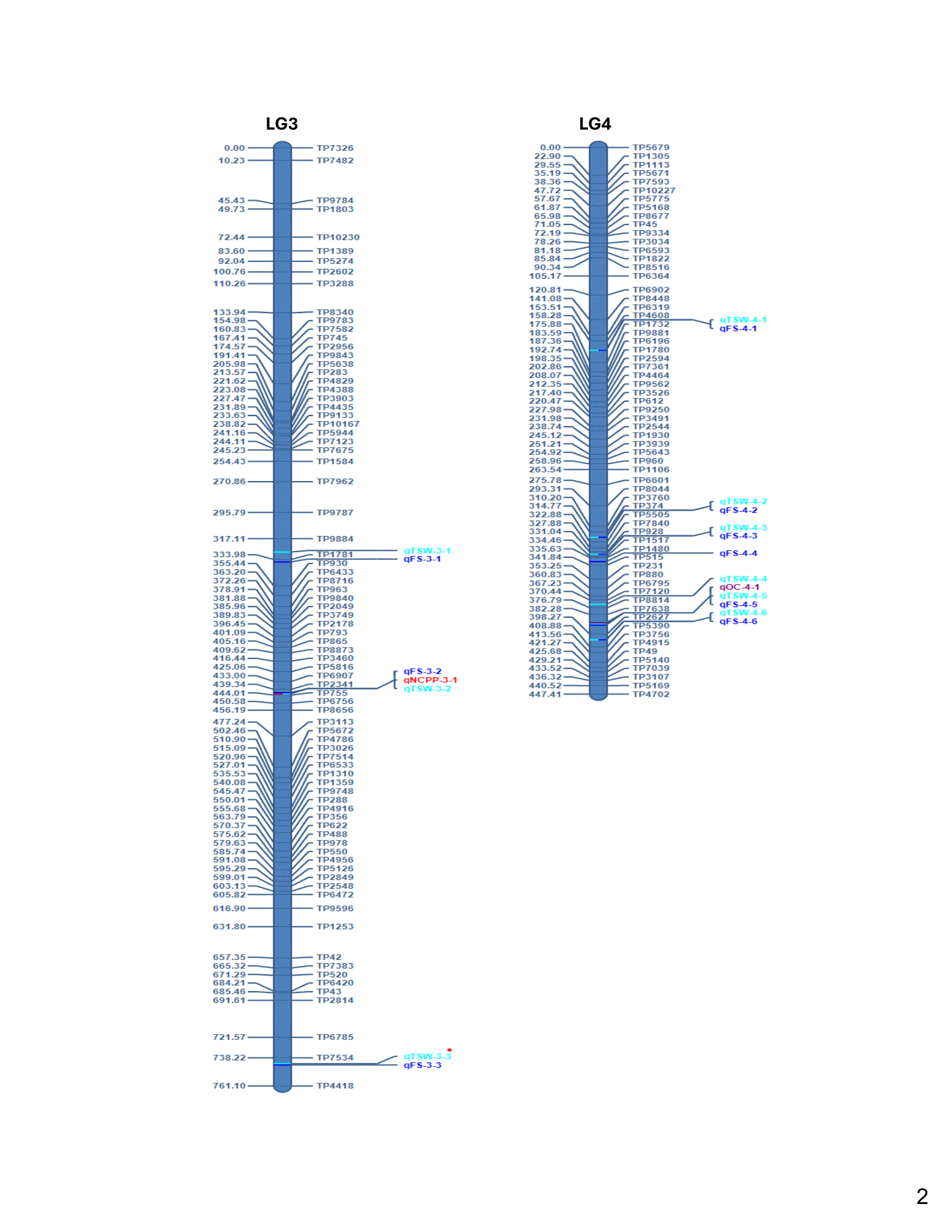

Supplement: Supplementary Figure 3 — Distribution of the 15 quantitative trait loci (QTL) across Guizotia abyssinica linkage groups for six quantitative characteristics: number of capitulum per plant (NCPP, red), number of seeds per plant (NSPP, green), thousand seed weight (TSW, turquoise), oil content (OC, purple), flower size (FS, blue), and days to flowering (DTF, yellow). [file DataSheet1.zip › Supplementary Figure S3/Supplementary Figure S3-2.tiff]

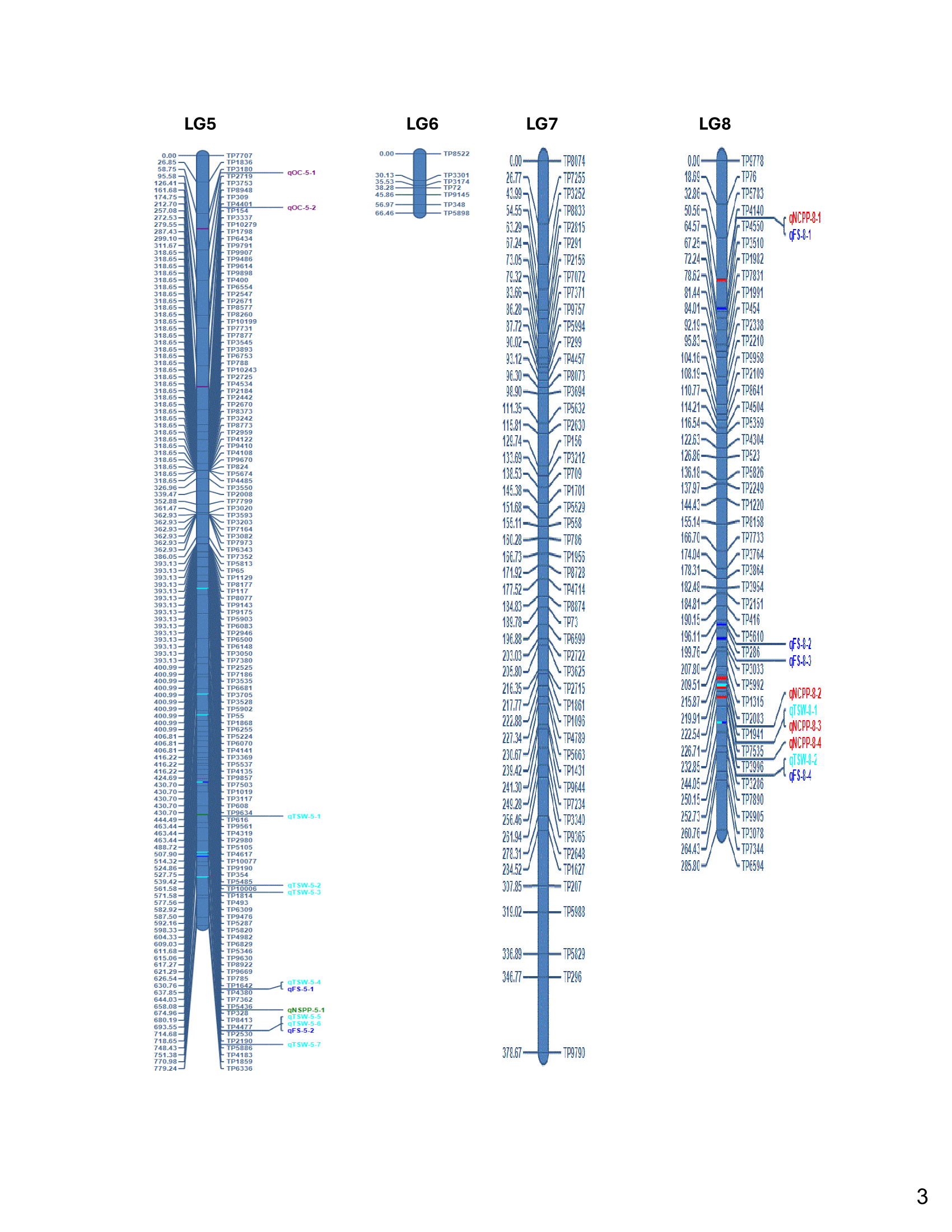

Supplement: Supplementary Figure 3 — Distribution of the 15 quantitative trait loci (QTL) across Guizotia abyssinica linkage groups for six quantitative characteristics: number of capitulum per plant (NCPP, red), number of seeds per plant (NSPP, green), thousand seed weight (TSW, turquoise), oil content (OC, purple), flower size (FS, blue), and days to flowering (DTF, yellow). [file DataSheet1.zip › Supplementary Figure S3/Supplementary Figure S3-3.tiff]

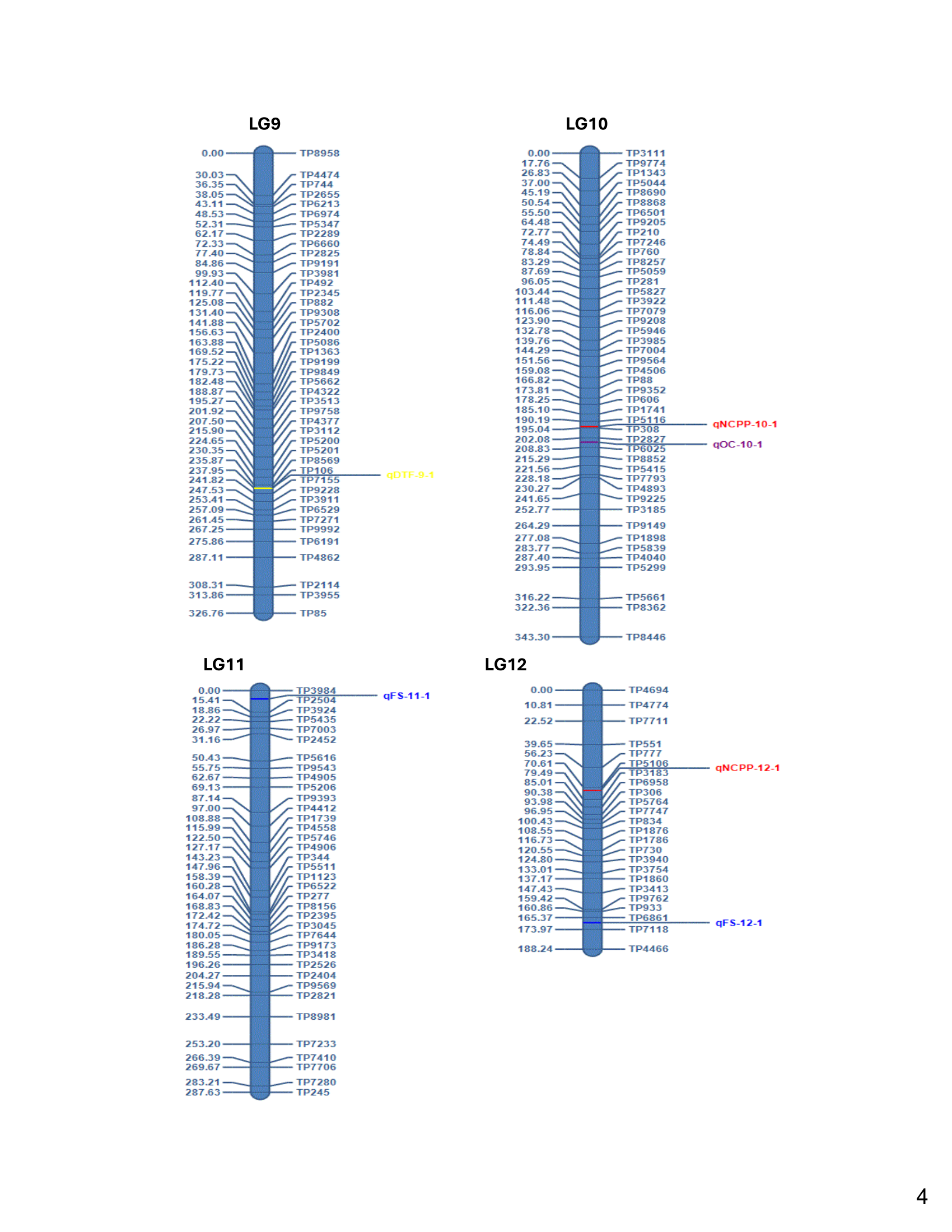

Supplement: Supplementary Figure 3 — Distribution of the 15 quantitative trait loci (QTL) across Guizotia abyssinica linkage groups for six quantitative characteristics: number of capitulum per plant (NCPP, red), number of seeds per plant (NSPP, green), thousand seed weight (TSW, turquoise), oil content (OC, purple), flower size (FS, blue), and days to flowering (DTF, yellow). [file DataSheet1.zip › Supplementary Figure S3/Supplementary Figure S3-4.tiff]

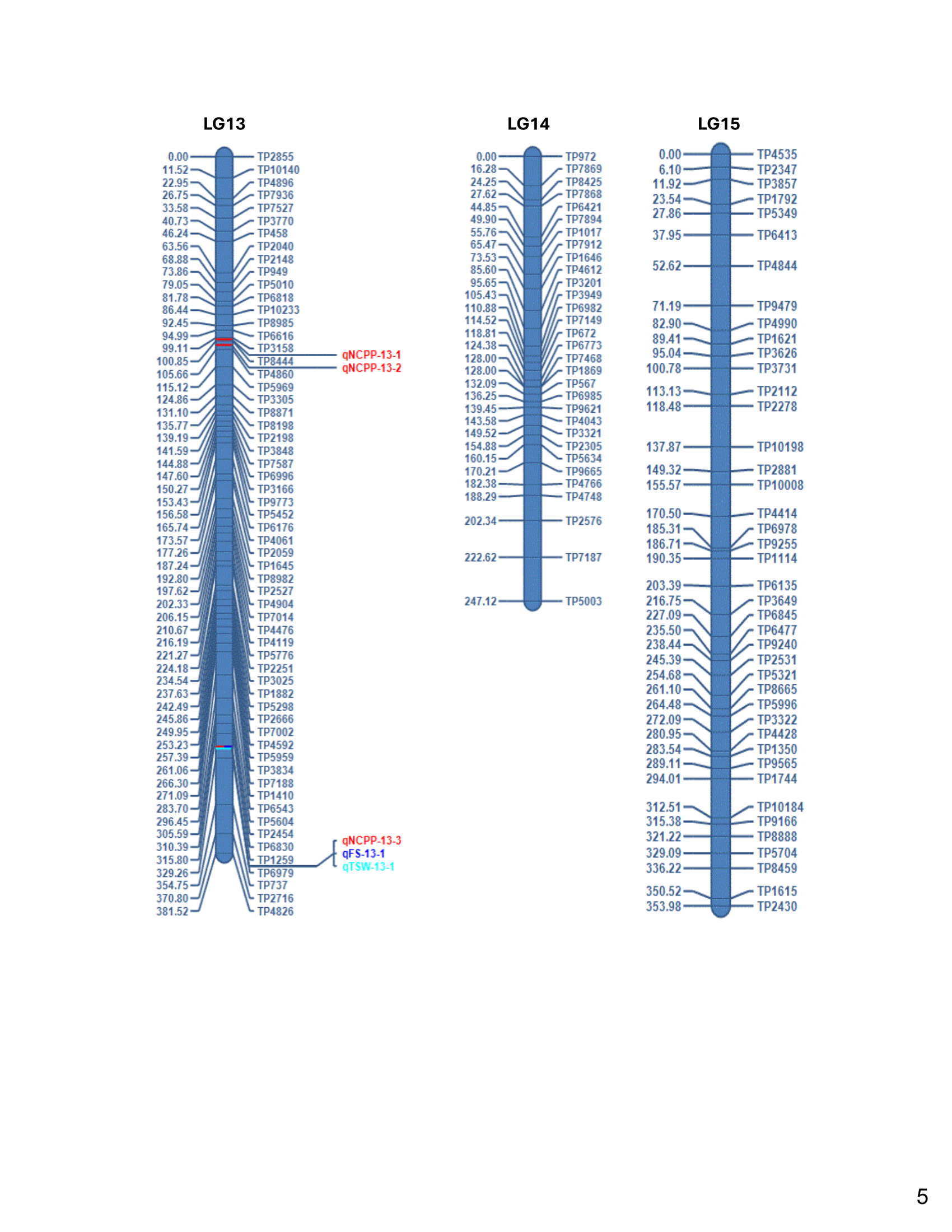

Supplement: Supplementary Figure 3 — Distribution of the 15 quantitative trait loci (QTL) across Guizotia abyssinica linkage groups for six quantitative characteristics: number of capitulum per plant (NCPP, red), number of seeds per plant (NSPP, green), thousand seed weight (TSW, turquoise), oil content (OC, purple), flower size (FS, blue), and days to flowering (DTF, yellow). [file DataSheet1.zip › Supplementary Figure S3/Supplementary Figure S3-5.tiff]
